# Statistical Approach to Crystal Nucleation in Glass-Forming Liquids

**DOI:** 10.3390/e23020246

**Published:** 2021-02-20

**Authors:** Joachim Deubener, Jürn W. P. Schmelzer

**Affiliations:** 1 Institute of Non-Metallic Materials, Clausthal University of Technology, Zehntner Str. 2a, 38678 Clausthal-Zellerfeld, Germany; 2 Institut für Physik der Universität Rostock, Albert-Einstein-Strasse 23-25, 18059 Rostock, Germany; juern-w.schmelzer@uni-rostock.de

**Keywords:** nucleation, crystal growth, general theory of phase transitions, glasses, glass transition, 64.60.Bd General theory of phase transitions, 64.60.Q- Nucleation, 81.10.Aj Theory and models of crystal growth, 64.70.kj Glasses, 64.70.Q- Theory and modeling of the glass transition, 65.40.gd Entropy

## Abstract

In this work, methods of description of crystal nucleation by using the statistical approach are analyzed. Findings from classical nucleation theory (CNT) for the average time of formation of the first supercritical nucleus are linked with experimental data on nucleation in glass-forming liquids stemming from repetitive cooling protocols both under isothermal and isochronal conditions. It is shown that statistical methods of lifetime analysis, frequently used in medicine, public health, and social and behavioral sciences, are applicable to crystal nucleation problems in glass-forming liquids and are very useful tools for their exploration. Identifying lifetime with the time to nucleate as a random variable in homogeneous and non-homogeneous Poisson processes, solutions for the nucleation rate under steady-state conditions are presented using the hazard rate and related parameters. This approach supplies us with a more detailed description of nucleation going beyond CNT. In particular, we show that cumulative hazard estimation enables one to derive the plotting positions for visually examining distributional model assumptions. As the crystallization of glass-forming melts can involve more than one type of nucleation processes, linear dependencies of the cumulative hazard function are used to facilitate assignment of lifetimes to each nucleation mechanism.

## 1. Introduction

Crystal nucleation in an undercooled glass-forming liquid is a stochastic process. Nucleation events in the bulk of the liquid (in the absence of heterogeneous nucleation cores) occur randomly in time and space and are independent from each other. Similarly, nucleation may proceed randomly in time at the given set of heterogeneous nucleation cores present in the liquid. In both cases, nucleation requires the transition via a thermodynamic potential barrier denoted also as work of critical cluster formation [1,2]. The choice of the thermodynamic potential appropriate for the description of nucleation (entropy, Helmholtz, or Gibbs free energy) depends on the thermodynamic constraints. The size of the critical cluster corresponds, in general, to a saddle point of the appropriate thermodynamic potential [3].

The formation of a critical cluster is forbidden by thermodynamic evolution laws; it is a stochastic process. In line with the statistical theory of fluctuation processes [4,5,6], the rate of formation of such critical clusters per unit time in a unit volume of a given ambient phase, *J*, can be expressed in a frequently sufficient approximation in the form

(1) $$J\left(T,p\right)=J_{0}\exp\left(-\frac{W_{c}}{k_{B}T}\right)\;,$$

as first suggested by Volmer and Weber [7]. Here, $k_{B}$ is the Boltzmann constant and *T* the absolute temperature. The pre-exponential term, $J_{0}$, reflects the kinetics of aggregation processes described containing, in particular, the appropriate diffusion coefficient or the viscosity of the liquid as a factor.

**Figure 1:**
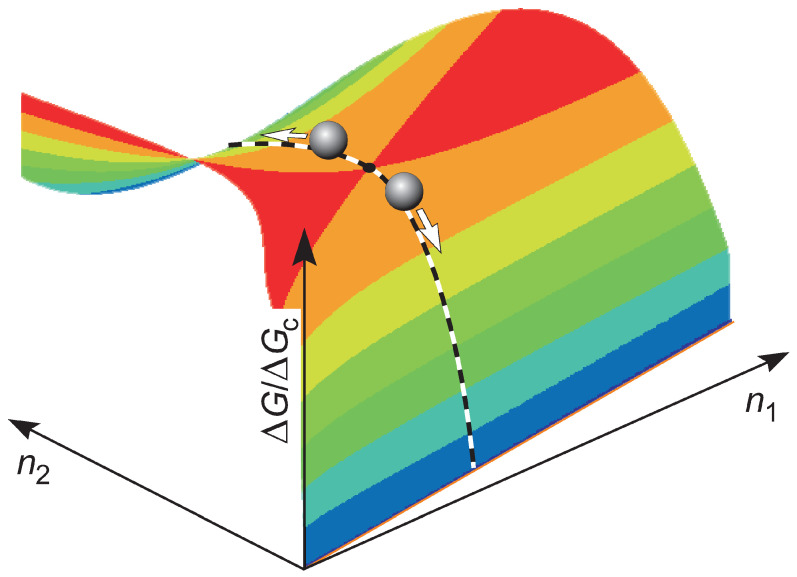
Change of the Gibbs free energy, ΔG, in crystal cluster formation (by $\Delta G_{c}$, its value at the critical cluster size is denoted). As an example, here the crystallites are supposed to consist of two components with the particles numbers $n_{1}$ and $n_{2}$.

An illustration of the nucleation process in terms of the standard model used widely in classical nucleation theory (CNT) is given in Figure 1. The main ideas retain its importance if modifications of this model have to be accounted for as discussed, e.g., long ago by J. J. Thomson in analyzing condensation on electrically charged particles, or more generally, treating phase formation processes under the influence of electric and magnetic fields [8] or other factors modifying the most probable pathways in nucleation (see, e.g., in [9,10]). In any case, once a cluster has been formed exceeding the size of the critical cluster, the further evolution to the crystalline phase proceeds in accordance with macroscopic thermodynamic evolution laws.

Utilizing CNT, the stochastic nature of crystal nucleation can be linked also with the width of the near-critical region in cluster size space, where the difference in the Gibbs energy of cluster formation is of the order of or less than $k_{B}T$ [11,12]. Under these conditions, no “driving force” is present in the transition region and it may take different times for the clusters to diffuse through this region in cluster size space. Thus, the determination of kinetic parameters such as the crystal nucleation rate relies on statistics.

In CNT, the statistical nature of nucleation is reflected only partly by measuring the average number of nuclei formed per unit time in a unit volume and interpreting it via Equation (1). However, other methods may result in a more explicit description of the stochastic nature of nucleation supplying us not only with average values, but with distributions in time and/or temperature of crystal nucleation events. For example, to maintain a higher level of statistical significance, data are frequently accessed using two different experimental approaches. The first covers deeply undercooled liquids, whereas the second deals with liquids at shallow undercooling. In this context, it is considered appropriate that the nose temperature of the TTT-diagram representations assigns the threshold value of undercooling. Thus, statistically sufficient data are achieved by analyzing ensembles of crystals when heating from temperatures below the glass transition temperature in a single run of one or two stages (deep undercooling) [13,14,15,16]. In turn, multiple runs of the same sample are necessary if the liquid is undercooled from above liquidus temperature (shallow undercooling). The latter technique—the so-called statistical approach—has its foundation from the fact that the first nucleated crystal grows fast and consumes the entire liquid volume shortly [17].

The statistical approach to crystal nucleation as an alternative to assessing nucleation kinetics via heat treatments has been developed over the past 50 years, starting with the analysis of the electrolytic deposition of cadmium on platinum single crystal electrodes in 1967 [18,19,20]. Later, further undercooled liquid metals, such as niobium and zircon [21], aluminum [22], gold and copper [23,24], and tin [25,26,27] were studied, but also water (pure and seeded) [28,29,30,31,32] and gas hydrates [33,34] were subjected to analysis of their crystal nucleation kinetics using repetitive cooling experiments of either isothermal or isochronal time-temperature protocols. In recent years, the approach was applied to the crystal nucleation of silicate melts using a conventional high-temperature differential scanning calorimeter (DSC) and a large number (usually >300) of successive cooling–heating cycles [35,36,37,38,39]. Meanwhile, it is also widely employed in molecular dynamics methods of modeling nucleation processes [40,41,42].

Time-to-event data are frequently analyzed by statistical methods [43] in a variety of applications. They play an important role in reliability engineering as well as for analysis of lifetime data in medicine, public health, social, and behavioral sciences. All disciplines share the subject to analyze the expected dwell time until a single event happens. The event under consideration covers a wide range, including the failure of a non-repairable component and the death of a human being. For materials engineering, mostly practical life cycle and performance predictions of a structural element or a finished component are developed, where the proportion of a test group that will survive (i.e., showing no failure) past a certain time and the rate at which the survivors will fail are predicted by statistical methods [44]. Statistical methods are also an established tool for lifetime predictions and mechanical strength analysis of glasses [45], but they are relatively rare for the prediction of critical cooling rates and crystal nucleation kinetics of glass-forming liquids [37].

In view of the above, and considering the increasing significance of statistical methods in glass technology (digitization of melting and forming processes), this study aims to provide the theoretical basis for the evaluation of time-to-event data generated by the application of statistical methods to crystal nucleation. Therefore, Section 2 presents the basic equations and combines interpretation in terms of the time of formation of the first supercritical cluster with those of lifetime analysis. First, the theoretical work considers isothermal nucleation and is extended then to nucleation at cooling, respectively, heating with a constant rate of change of temperature. Section 3 of this study presents some applications for lifetime data with emphasis to the contact behavior of undercooled silicate melts with metals, i.e., heterogeneous crystallization either at constant temperature or at cooling with a constant rate. A summary of the results and their discussion (Section 4) and of the conclusions (Section 5) completes the paper.

## 2. Statistical Approach to Crystal Nucleation

### 2.1. Basic Equations

As discussed in detail in the introduction, the formation of crystal nuclei in a metastable liquid is a random process. Here, we briefly outline the basic results for its theoretical description in such terms following first widely the methods as presented in [42,46,47] and extending them in a second step relying on general statistical methods of lifetime analysis.

The probability, $\tilde{\omega }\left(\tau \right)$, of formation of the first critical crystallite in the sample in the small time interval, Δτ, between τ and τ+Δτ can be expressed generally in the form [46]

(2) $$\tilde{\omega }\left(\tau \right)=\lambda \left(T,p,\tau \right)\Delta \tau \;.$$

The probability of absence of such processes in that particular time interval between τ and τ+Δτ is consequently given by $\left(1-\tilde{\omega }\right)$. In Equation (2), the parameter λ is the mean rate of formation of critical nuclei. It may depend on temperature, *T*; pressure, *p*; and also explicitly on time, τ. If λ is a constant, then the process is a Poisson stochastic process.

The probability, $P_{m}\left(\tau +\Delta \tau \right)$, that at the moment of time, τ+Δτ, *m* critical clusters are present in the system formed in the course of nucleation processes starting at τ=0 is given, consequently, by

(3) $$P_{m}\left(\tau +\Delta \tau \right)=\left(1-\lambda \Delta \tau \right)P_{m}\left(\tau \right)+\left(\lambda \Delta \tau \right)P_{m-1}\left(\tau \right)\;.$$

Here, the number of critical clusters, *m*, can have the values m=0,1,2,… We assume that at τ=0 critical clusters do not exist in the system (m=0), the initial conditions can be written therefore in the form

(4) $$P_{m}\left(0\right)=\left\{\begin{array}{ccc} 1 & \mathrm{for} & m=0\\ 0 & \mathrm{for} & m=1,2,\ldots \end{array}\right.\;.$$

Here, $P_{0}\left(\tau \right)$ is the probability of absence of critical clusters in the system under consideration. A Taylor expansion of Equation (3) truncated at linear in Δτ terms results in

(5) $$\frac{dP_{0}\left(\tau \right)}{d\tau }=-\lambda P_{0}\left(\tau \right)\;,$$

(6) $$\frac{dP_{m}\left(\tau \right)}{d\tau }=-\lambda P_{m}\left(\tau \right)+\lambda P_{m-1}\left(\tau \right)\;,\;m=1,2,\ldots \;.$$

The solution of Equations (4)–(6) supplies us with the desired probabilities of formation of m=1,2,… critical clusters, $P_{m}\left(\tau \right)$, respectively, the probability of their absence, $P_{0}\left(\tau \right)$, in dependence on time, τ. In the subsequent analysis, we will concentrate mainly on the probability of formation of the first critical cluster in the system under consideration. In line with above given equations, the density of probability, ω(τ), of formation of the first critical cluster per unit time is given generally by

(7) $$\omega \left(\tau \right)=\lambda P_{0}\left(\tau \right)\;.$$

### 2.2. Nucleation at Constant Temperature and Pressure

#### 2.2.1. Interpretation in Terms of Times of Formation of the First Supercritical Nucleus

Pressure and temperature are assumed to be fixed here first. Provided the density of probability of formation of a critical nucleus does not depend also on time (λ= constant, i.e., steady-state nucleation conditions are established in the system under consideration), the solutions of Equations (4)–(6) are given by [46]

(8) $$P_{0}\left(\tau \right)=\exp\left(-\lambda \tau \right)\;,$$

(9) $$P_{1}\left(\tau \right)=\lambda \tau \exp\left(-\lambda \tau \right)\;,$$

(10) $$P_{m}\left(\tau \right)=\frac{{\left(\lambda \tau \right)}^{m}}{m!}\exp\left(-\lambda \tau \right)\;,\;m=2,3,\ldots \;.$$

In such continuous description, the density of probability of formation of the first critical nucleus at time, τ, is given in line with Equation (7) as

(11) ω(τ)=λexp(−λτ).

Consequently, the part of the number of clusters formed in the time interval, τ,τ+dτ, in such continuous description is given by ω(τ)dτ and the average time of formation of the first supercritical nucleus at the considered here steady-state conditions, ${\langle \tau \rangle }_{ss}$, can be expressed as

(12) $${\langle \tau \rangle }_{ss}=\int_{0}^{\infty }\omega \left(\tau \right)\tau d\tau =\int_{0}^{\infty }\tau \lambda \exp\left(-\lambda \tau \right)d\tau =\frac{1}{\lambda }\;.$$

We will consider here first the situation that the rate of growth of a supercritical cluster is sufficiently large so that the characteristic time of evolution of a critical cluster to observable sizes is small as compared with the typical times of nucleation, in particular, with the average time of formation of the first critical nucleus. Consequently, the time of formation of the first critical nucleus determines the time of crystallization of the liquid. At such conditions, Equation (11) can be directly employed for a comparison with experimental data determining the number, $n_{i}\left(\tau_{i}\right)$, of cases that the first supercritical nucleus is formed in $N_{0}$ experiments in the time interval $\tau_{i},\tau_{i}+\Delta \tau$. More precisely, if $n_{i}\left(\tau_{i}\right)$ is the number of observations of the first supercritical nuclei in the interval $\left(\tau_{i},\tau_{i}+\Delta \tau \right)$ and $N_{0}$ is the total number of experiments where the formation of the first supercritical nucleus is observed, then we can write in accordance with Equations (9) and (11)

(13) $$\frac{n_{i}\left(\tau_{i}\right)}{N_{0}}=\Omega \left(\tau_{i}\right)\;,\;\Omega \left(\tau \right)=\omega \left(\tau \right)\Delta \tau =\lambda \exp\left(-\lambda \tau \right)\Delta \tau \;.$$

The average time of formation of the first supercritical nucleus can be expressed in such discrete formulation in analogy to Equation (12) as

(14) $${\langle \tau \rangle }_{ss}=\sum_{i}^{f}\left(\frac{n_{i}}{N_{0}}\right)\tau_{i}\;.$$

Here, *f* is the number of intervals the nucleation period is divided into.

In CNT, the change of the average number of critical clusters, *n*, per unit volume is given by the nucleation rate, *J*, defined as the average number of critical clusters formed per unit time in a unit volume [11,12,42,46,47,48]. The change of the average number of supercritical clusters, n(t), in a volume, *V*, of the liquid, can be written at the considered conditions (constancy of pressure and temperature) consequently as [11,12,46]

(15) $$J=\frac{1}{V}\frac{dn}{dt}\;,\;n\left(t\right)=\int_{0}^{t}JVdt\;.$$

The average time of formation of the first supercritical nucleus (n=1) can be expressed in such treatment in the form

(16) $$\int_{0}^{\langle \tau \rangle }JVdt=1\;.$$

At steady-state conditions (J= constant), we obtain as a special case

(17) $${\langle \tau \rangle }_{ss}=\frac{1}{JV}\;.$$

A comparison with Equation (12) yields

(18) λ=JV.

With such choice of the parameter λ, one arrives at an agreement of experimental data (Equation (13)) with theoretical predictions (Equation (11)) with respect to the distribution of crystal nucleation events or its probability in dependence on time. An example for the typical picture obtained in the application of the method of determination of λ in steady-state nucleation at fixed boundary conditions in comparison of experimental data (Equation (13)) with theoretical predictions (Equation (11)) for the times of formation of the first supercritical nucleus can be found in the monograph by Skripov and Koverda (Figure 53 in [46]). The application of this method for the interpretation of own experimental data we will illustrate here later in Figure 3.

#### 2.2.2. Interpretation in Terms of Lifetime Analysis

In the alternative but equivalent approach of lifetime analysis, we may take the sum of the terms $\left(n_{i}/N_{0}\right)$ from i=1,2,…,k. Equation (13) then yields

(19) $$\tilde{P_{1}}\left(\tau \right)=\sum_{i=1}^{k}\frac{n_{i}\left(\tau \right)}{N_{0}}=1-P_{0}\left(\tau \right)=1-\exp\left(-\lambda \tau \right)\;.$$

Here, τ specifies the time at which the first *k* observations of critical nuclei have been performed and $\tilde{P_{1}}\left(\tau \right)$ is the probability that the first critical cluster is formed until that moment of time. Equation (19) can be expressed in the form

(20) $$\tilde{H}\left(\tau \right)=\ln\left[\frac{1}{1-\sum_{i=1}^{k}\frac{n_{i}\left(\tau \right)}{N_{0}}}\right]=\lambda \tau \;.$$

Here, *k* may have different suitable values in the range k=1,2,…,f, where *f* is the number of discrete time intervals at which nucleation is observed. Equation (20) allows one to determine the parameter λ plotting the function $\tilde{H}$ in dependence on τ. Of course, the methods of determination of the parameter λ via Equations (14) and (18), respectively, Equation (20) have to coincide. Having obtained the value of λ, the probability of formation of the first critical cluster, $\tilde{P_{1}}\left(\tau \right)$, in the time interval from τ=0 till τ is given by Equation (19).

We can reformulate these relations identifying the number of intervals of measurements with the number of successful measurements, $N_{0}$. Then, in each of the time intervals one critical cluster is formed, each of the numbers $n_{i}$ is equal to one ($i=1,2,\ldots ,N_{0}$) and $\tilde{P_{1}}\left(\tau \right)$ defines the lifetime distribution function, F(τ). The inverse probability, which is called survival function S(τ), is then the probability that events have not been occurred until that moment of time,

(21) $$F\left(\tau \right)=\tilde{P_{1}}\left(\tau \right)=1-S\left(\tau \right)\;,$$

(22) $$S\left(\tau \right)=1-\tilde{P_{1}}\left(\tau \right)\;.$$

In mechanical survival problems, F(τ) and S(τ) are called failure function and reliability function (denoted also by R(τ)), respectively. Further we define f(τ) as the derivative of F(τ). It expresses the density function of the lifetime distribution (named as failure density function in mechanical survival analysis)

(23) $$f\left(\tau \right)=\frac{dF(\tau )}{d\tau }\;.$$

f(τ) represents the event rate per unit time.

In the analysis of the problems under consideration, the hazard function, h(τ), is widely employed. It is defined as

(24) $$h\left(\tau \right)=\frac{f(\tau )}{S(\tau )}=\frac{f(\tau )}{1-F(\tau )}=\frac{d}{d\tau }\ln\left(1-F(\tau )\right)=-\frac{d}{d\tau }\ln\left(S(\tau )\right)\;.$$

It is the main object of interest not only in reliability engineering (see bathtub curve of the failure rate [44]), but also for the here analyzed survival times of crystal nucleation. h(τ) defines the event rate at time τ based on the condition of survivorship until time τ or later. Thus, h(τ) is the instantaneous failure rate or death rate of survivors aged by τ. By analogy, the cumulative hazard function H(τ), defines the accumulation of the hazard over time,

(25) $$H\left(\tau \right)=\int_{0}^{\tau }h\left(\tau \right)d\tau \;.$$

It is related to the survival function through the expression

(26) $$H\left(\tau \right)=-\ln\left[S\left(\tau \right)\right]\;.$$

With Equations (19), (21), and (22) one obtains in analogy to Equation (20)

(27) $$\tilde{H}\left(\tau \right)=\ln\left[\frac{1}{1-\sum_{i=1}^{k}\frac{n_{i}\left(\tau \right)}{N_{0}}}\right]\;,$$

where *k* may have the values $k=1,2,\ldots ,N_{0}$.

It will be shown in the following sections that the cumulative hazard plotting can also be used to test the homogeneity of the underlying Poisson process. As the plotting positions $\tilde{H}\left(\tau \right)$ of Equation (27) are calculated independently of the Poisson model, a reasonable straight-line fit to the points can confirm that the chosen model and the data are consistent. In case of fixed temperature and pressure, we can expect such a homogeneous Poisson process, which is expressed by the exponential distribution (cf. Equations (8)–(11)):

(28) $$f\left(\tau \right)={\lambda e}^{-\lambda \tau }$$

and

(29) $$F\left(\tau \right)=\int_{0}^{\tau }f\left(\tau \right)d\tau =1-e^{-\lambda \tau }\;.$$

Then, the hazard functions are

(30) $$h\left(\tau \right)=\frac{f(\tau )}{1-F(\tau )}=\frac{{\lambda e}^{-\lambda \tau }}{{1-(1-e}^{-\lambda \tau })}=\lambda$$

and

(31) $$H\left(\tau \right)=\lambda \tau$$

in line with the result given in Equation (20).

In the comparison of experimental data with theoretical predictions as performed so far, it is assumed that the rate of growth of supercritical clusters is sufficiently large so that the time required to grow to observable sizes can be neglected as compared to the typical times of nucleation. Moreover, so far we neglected time-lag effects in nucleation. The effect of time-lag and finite times of growth of the critical clusters to experimentally observable sizes on the method of comparison of theoretical treatment and experimental data will be analyzed in the next sections.

### 2.3. Discussion of Possible Limits of Applicability of the Theoretical Approach

#### 2.3.1. Account of Time-Lag Effects

As noted first by Zeldovich [49], a certain time, $\tau_{ns}$, denoted as time-lag in nucleation, is required to establish steady-state nucleation rates and steady-state cluster-size distributions for clusters up to critical sizes in a given system after it has been transferred to the desired metastable initial state. Following Zeldovich, the nucleation rate, $J_{ns}$, in this initial non-steady stage is described as

(32) $$J_{ns}\left(T,p;t\right)=J\left(T,p\right)\exp\left(-\frac{\tau_{ns}}{t}\right)\;.$$

Extensions of the original concepts of Zeldovich are reviewed in [11,12,42,50].

Accounting for such non-steady-state conditions, the parameter λ introduced with Equation (2) is not a constant and Equations (8)–(11) are, in general, not valid any more. A detailed analysis of how the theoretical approach has to be modified, accounting for time-lag effects on nucleation, is presented in [42]. In particular, utilizing Equation (32), Equations (9) and (11) get the form

(33) $$P_{0}\left(\tau \right)=\exp\left(-JV\int_{0}^{\tau }\exp\left(-\frac{\tau_{ns}}{t}\right)dt\right)\;,$$

(34) $$\omega \left(\tau \right)=JV\exp\left(-\frac{\tau_{ns}}{\tau }\right)\exp\left(-JV\int_{0}^{\tau }\exp\left(-\frac{\tau_{ns}}{t}\right)dt\right)\;.$$

For the specification of these functions, consequently, the knowledge is required of how the steady-state nucleation rate is approached. However, the analysis shows that independent of the course of approach of steady-state conditions, the method developed in Section 2.2 retains its applicability if the analysis is retained to times when steady-state conditions are established.

Here, we will illustrate this main result employing an approximation for Equation (32) of the form

(35) $$J_{ns}\left(T,p;t\right)=\left\{\begin{array}{ccc} 0 & \mathrm{at} & 0\leq t<t_{ind}\\ \; & \; & \\ J(T,p) & \mathrm{at} & t_{ind}\leq t \end{array}\right.\;.$$

The induction time, $t_{ind}$, can be identified in a good approximation with the time-lag in nucleation, $\tau_{ns}$ [11,12,16,42]. Employing this approximation, crystal nucleation does not occur at all in the time interval $0\leq t\leq t_{ind}$. The analysis of nucleation processes for the times $t>t_{ind}$ can be performed again in the same way as described in Section 2.1 and Section 2.2. Merely, now, τ=0 has to be identified with $t=t_{ind}$. We can, consequently, employ the methods as described earlier utilizing only the results for the part of the nucleation process where steady-state conditions are established. As noted, this consequence can be derived also in a strong analysis as performed in [42].

#### 2.3.2. Account of Finite Times of Growth of Critical Clusters to Observable Sizes

Critical crystallites are, as a rule, too small to be observed with standard experimental techniques. For this reason, in the experimental determination of the times of formation of critical clusters, one has to account also for the typical times of evolution of the critical clusters to measurable sizes. As shown in detail in [42,48], the average time, $\langle t_{ex}\rangle$, required for a first-formed critical cluster to evolve to experimentally observable sizes (the incubation time [11,12,16,42,48], sometimes also denoted as lag-time [29,36]) can be expressed as

(36) $$\langle t_{ex}\rangle =t_{ind}+{\langle \tau \rangle }_{ss}+t_{growth}\;.$$

The only effect of time-lag and growth of the supercritical cluster to observable sizes is that the experimentally observed distributions $n_{i}\left(\tau \right)/N$ are shifted to larger times. In order to obtain the average time of formation of a critical cluster, we have to define τ as

(37) $$\tau =t_{ex}-t_{ind}-t_{growth}\;.$$

With such redefinition of the time scales, we will arrive at a coincidence of theoretical and experimental curves. The part of the experimental curves connected with non-steady state nucleation is excluded, the growth to observable sizes leads merely to a shift in time of the observation of nucleation. Both factors, consequently, do not affect the results for the determination of the average time in steady-state nucleation. In such approach, there is no need to determine the values of the induction time and the time of growth to experimentally measurable sizes, one has merely to identify τ=0 in Equation (11) with the maximum of the distributions, $\left(n_{i}/N_{0}\right)$ (Equation (13)), observed experimentally.

### 2.4. Nucleation at Cooling or Heating with a Constant Rate of Change of Temperature

#### 2.4.1. Interpretation in Terms of Times of Formation of the First Supercritical Nucleus

We will advance the theory here for the case, again, that steady-state conditions are established formulating the general relations for nucleation in cooling or heating. In more detail, we will consider crystallization in cooling starting at the melting temperature.

Assuming that the temperature of the liquid is changed with a rate,

(38) $$q=\frac{dT}{d\tau }=\dot{T}\;,$$

the basic equations for the description of crystal nucleation, in particular, the relations for the probability of absence of critical crystals, $P_{0}\left(\tau \right)$, and the density of probability of evolution of the first critical nucleus, ω(τ), are given by

(39) $$P_{0}\left(\tau \right)=\exp\left(-\int_{0}^{\tau }\lambda \left(\tau^{\prime }\right)d\tau^{\prime }\right)\;,$$

(40) $$\omega \left(\tau \right)=\lambda \left(\tau \right)P_{0}\left(\tau \right)=\lambda \left(\tau \right)\exp\left(-\int_{0}^{\tau }\lambda \left(\tau^{\prime }\right)d\tau^{\prime }\right)\;.$$

For any temperature, $T_{in}$, of the initial state, Equation (38) defines uniquely the dependence T=T(τ) both for cooling and heating processes. In terms of temperature, we can reformulate Equation (40) as

(41) $$\omega \left(T\right)=\left\{\begin{array}{cc} -\frac{J(T)V}{\dot{T}}\exp\left(-\int_{T_{in}}^{T}J\left(T^{\prime }\right)V\frac{dT^{\prime }}{\dot{T}}\right) & \mathrm{at}\;\mathrm{cooling}\\ \frac{J(T)V}{\dot{T}}\exp\left(-\int_{T_{in}}^{T}J\left(T^{\prime }\right)V\frac{dT^{\prime }}{\dot{T}}\right) & \mathrm{at}\;\mathrm{heating} \end{array}\right..$$

These general relations will now be employed for the analysis of nucleation at cooling with a constant rate of change of temperature.

For the cooling process we start at the melting temperature, $T_{m}$. With Equation (41) we arrive immediately at

(42) $$\omega \left(T\right)=-\frac{J(T)V}{\dot{T}}\exp\left(-\int_{T_{m}}^{T}J\left(T^{\prime }\right)V\frac{dT^{\prime }}{\dot{T}}\right)$$

or [46]

(43) $$\omega \left(T\right)=-\frac{J(T)V}{\dot{T}}\exp\left(-\frac{V}{\dot{T}}\int_{J(T_{m})}^{J(T)}\frac{dJ}{d\ln J/dT}\right)\;.$$

Further, as the derivative of the logarithm of the steady-state nucleation rate depends only weakly on temperature, Equation (43) may be transformed into

(44) $$\omega \left(T\right)=-\frac{J(T)V}{\dot{T}}\exp\left(-\frac{J(T)V}{\dot{T}d\ln J/dT}\right)\;.$$

Here, it is also accounted for that the nucleation rate equals zero at $T=T_{m}$. It is supposed here further that we remain inside the range of temperatures where the nucleation rate decreases with increasing temperature.

Similarly to Equation (13), we can now compare experimental data of the frequency of occurrence of the first critical nucleus in the time interval, $\tau_{i},\tau_{i}+\Delta \tau$, respectively, the temperature interval, $T_{i},T_{i}-\delta T$ (δT>0), with the theoretical predictions. For the case under consideration, we obtain

(45) $$\frac{n_{i}\left(T_{i}\right)}{N_{0}}=\Omega \left(T_{i}\right)\;,$$

(46) $$\Omega \left(T\right)=\omega \left(T\right)\delta T=-\frac{J(T)V}{\dot{T}}\exp\left(-\frac{J(T)V}{\dot{T}d\ln J/dT}\right)\delta T\;,\;\delta T>0\;.$$

The typical course of the dependence of Ω(T) (Equation (45)), respectively, the density of probability of evolution of the first critical nucleus, ω(T) (Equation (44)), on undercooling, $\Delta T=T_{m}-T$, in steady-state nucleation at cooling with a constant rate of change of temperature is shown on Figure 56 in the monograph by Skripov and Koverda [46] and here in the analysis of own data in Figure 7. It has a maximum at the temperature $T=T_{max}$. Employing the approximation introduced in the transformation of Equation (43) into Equation (44), this maximum is defined via

(47) $$J\left(T_{max}\right)V=\dot{T}{\left.\frac{d\ln J}{dT}\right|}_{T=T_{max}}\;.$$

In analogy to the average time of formation of the first supercritical nucleus at steady-state conditions and constant values of pressure and temperature, ${\langle \tau \rangle }_{ss}=1/\left(JV\right)$ (Equation (17)), we may define a characteristic time of formation of the first critical nucleus in cooling with a constant rate as

(48) $${\langle \tau \rangle }_{q}=\frac{1}{J(T_{max})V}\;.$$

In accordance with Equation (47), it can be expressed as

(49) $${\langle \tau \rangle }_{q}={\left(\dot{T}{\left.\frac{d\ln J}{dT}\right|}_{T=T_{max}}\right)}^{-1}\;.$$

The average undercooling, ${\langle \Delta T\rangle }_{q}$ ($\Delta T=T_{m}-T$), at which the first critical crystallite is formed at a given constant rate of cooling, can be expressed, consequently, as

(50) $${\langle \Delta T\rangle }_{q}=-{\left({\left.\frac{d\ln J}{dT}\right|}_{T=T_{max}}\right)}^{-1}\;.$$

As mentioned, we remain inside the range of temperatures where the nucleation rate decreases with increasing temperature. This is the origin of the minus sign in Equation (50). Note, moreover, that in above derived relations, Equations (41)–(50), for *J* the appropriate expressions for the steady-state nucleation rate in dependence on temperature have to be accounted for. Corrections connected with the account of athermal nucleation (cf. [11,12]) may be neglected as we consider here the case of formation of the first supercritical nucleus.

As evident from Equations (47)–(50), the average time of appearance of the first supercritical nucleus depends on the cooling rate, the sample size, and the temperature dependence of the steady-state nucleation rate as confirmed by experiment (see, e.g., in [23,27,51,52,53,54]). These relations will be employed here in the interpretation of experimental data in heterogeneous nucleation in cooling. For such cases, it can be supposed that nucleation proceeds in the steady-state regime. This condition is satisfied as near to the melting temperature and, down to temperatures corresponding to the maximum of the steady-state nucleation rate, the time-lag in nucleation is negligibly small as compared with the average time of formation of the first supercritical nucleus at steady-state conditions [42,48]. Furthermore, the rate of growth is sufficiently large and corrections accounting for finite times of growth of critical clusters to measurable sizes can be neglected, as a rule.

Time-lag effects and effects of finite times of growth of the clusters to experimentally measurable sizes we consider as important for the case of determination of the probability of occurrence of the first critical cluster in heating starting from a low temperature where the rate of crystal nucleation tends to zero due to low values of the diffusion coefficient governing nucleation, respectively, very large values of the viscosity of the liquid. Here, a variety of problems have to be solved in order to arrive at appropriate relations for determining the characteristic parameters of crystal nucleation [55,56,57]. As it seems, in order to interpret experimental data in such case, a theoretical treatment of nucleation based on the solution of the set of kinetic equations for the average rate of crystal nucleation and growth (see, e.g., in [11,12,46,58]) is preferable. In any case, heating a dependence of the probability of crystal nucleation on ΔT can also be expected to be of the form as obtained for cooling processes. It follows that, both in cooling and heating with a constant rate of change of temperature, the same typical features of crystallization processes are observed as described in an alternative form via the Kissinger equation widely employed and discussed in materials science [59,60,61]. In this way, the statistical approach to nucleation gives a foundation of some main conclusions obtained by the Kissinger equation and may resolve also some problems in its application. Indeed, while, with respect to the formation of the first critical nuclei, the situation is similar, it is, as a rule, different with respect to crystal growth. The maxima of the growth rates are, as a rule, located at higher temperatures as compared with the maxima of the nucleation rates [62,63] resulting in differences with respect to the possibility of the critical nuclei to grow to macroscopic sizes. However, a detailed discussion of this circle of problems goes beyond the scope of the present paper.

As mentioned already in the introduction, the statistical treatment supplies us with a more detailed treatment of crystal nucleation as compared with CNT. As one particular result, it supplies us with the experimental determination of the dependence of the steady-state nucleation rate on temperature intensively studied in CNT. This dependence is also required in order to interpret experimental results on crystal nucleation at given rates of change of temperature. Overviews on well-established methods and new results in the theoretical description of crystal nucleation in terms of CNT can be found, e.g., in [11,12,64,65,66]. Note also that pressure-induced nucleation [67,68,69] can be treated similarly to nucleation caused by changes of temperature both for nucleation at constant pressure and nucleation at constant rates of change of pressure as recently demonstrated in [70,71,72].

#### 2.4.2. Interpretation in Terms of Lifetime Analysis

At the experimental conditions analyzed, crystal nucleation occurs at different temperatures from run to run. Such a process has all the properties of a Poisson process (nucleation occurs randomly in time and temperature), except for the fact that its rate is a function of temperature, i.e., λ=λ(T). This generalization is denoted as non-homogeneous Poisson process.

The cost of this generalization is that the specific function by which λ varies with temperature has to be defined for each type of crystal nucleation process. Firstly and in analogy to Equation (19) and accounting for Equations (22), (39), and (41), we can estimate $\tilde{P_{1}}\left(T\right)$ by taking the sum of the terms $\left(n_{i}/N_{0}\right)$

(51) $$\tilde{P_{1}}\left(T\right)=\sum_{i=1}^{k}\frac{n_{i}\left(T_{i}\right)}{N_{0}}=1-\exp\left(-\int_{T_{in}}^{T}J\left(T^{\prime }\right)V\frac{dT^{\prime }}{\dot{T}}\right)\;,$$

(52) $$S\left(T\right)=\exp\left(-\int_{T_{in}}^{T}J\left(T^{\prime }\right)V\frac{dT^{\prime }}{\dot{T}}\right)\;,$$

For the estimate of the cumulative hazard, H(τ), we have with Equation (26)

(53) $$H\left(T\right)\;=\ln\left[\frac{1}{1-\sum_{i=1}^{k}\frac{n_{i}\left(T_{i}\right)}{N_{0}}}\right]=\int_{T_{in}}^{T}J\left(T^{\prime }\right)V\frac{dT^{\prime }}{\dot{T}}\;.$$

Here, we will consider again cooling starting at the melting temperature. Employing the same approximation as in the transformation of Equation (42) into Equation (43), we arrive at

(54) $$H\left(T\right)\;=\ln\left[\frac{1}{1-\sum_{i=1}^{k}\frac{n_{i}\left(T_{i}\right)}{N_{0}}}\right]=\frac{J(T)V}{|\dot{T}\left||d\ln J/dT\right|}\;.$$

In accordance with Equation (47), at the temperature $T_{max}$, corresponding to a maximum of Ω(T) (Equation (45)), respectively, the density of probability of evolution of the first critical nucleus, ω(T) (Equation (44)), the function H(T) approaches one, i.e., $H\left(T_{max}\right)=1$. Consequently, the cumulative hazard function can be expressed as

(55) $$H\left(T\right)\;≅H\left(T_{max}\right)+{\left.\frac{d}{dT}\left(\frac{J(T)V}{|\dot{T}\left||d\ln J/dT\right|}\right)\right|}_{T=T_{max}}\left(T-T_{max}\right)\;,$$

or

(56) $$H\left(T\right)\;≅1-{\left.\frac{J(T)V}{|\dot{T}|}\right|}_{T=T_{max}}\left(T-T_{max}\right)\;.$$

In the derivation of Equation (56), we again employed the approximation that the derivative of the logarithm of the steady-state nucleation rate depends only weakly on temperature. Considering also the rate of change of temperature, $|\dot{T}|$, and the volume, *V*, as constant, we may write

(57) $$\frac{d}{dT}\left(\frac{J(T)V}{|\dot{T}\left||d\ln J/dT\right|}\right)\;≅\;\left(\frac{V}{|\dot{T}\left||d\ln J/dT\right|}\right)\frac{dJ(T)}{dT}≅-\frac{J(T)V}{|\dot{T}|}\;,$$

as (dJ/dT) has a negative sign.

The slope of the cumulative hazard function is consequently determined by the steady-state nucleation rate at $T=T_{max}$; the volume of the liquid, *V*; and the cooling rate, $|\dot{T}|$. Alternatively, as the derivative of the logarithm of the steady-state nucleation rate depends only weakly on temperature, Equations (47) and (54) yield

(58) $$H\left(T\right)=\ln\left[\frac{1}{1-\sum_{i=1}^{k}\frac{n_{i}\left(T_{i}\right)}{N_{0}}}\right]≅\frac{J(T)}{J(T_{max})}\;.$$

Generally, crystallization of glass-forming melts can involve more than one type of heterogeneous nucleation processes, as the same or different crystal phases can nucleate simultaneously in the volume or different sites at external surfaces can be active. The respective relations can be formulated appropriately in terms of classical nucleation theory. The temperature dependence of the steady-state nucleation rate can be expressed in a frequently good approximation as [11,12,46]

(59) $$J\left(T\right)=\sum_{j}\left(\frac{C_{j1}}{\eta }\right)\exp\left(-\frac{C_{j2}}{T{\left(\Delta T\right)}^{2}}\right)\;,$$

where *j* denotes the number of concurrent nucleation processes. Modifications of this relation can be advanced accounting for more correct expressions for the thermodynamic driving force [73] going beyond the Tammann–Meissner–Rie equation, the decoupling of diffusion and viscosity [74] or accounting for different mechanisms of how heterogeneous nuclei may act on crystal nucleation [23,27,51,52,53,54].

Following the statistical analysis, we can use for each nucleation process the hazard estimator $H\left(T\right)$ to derive the plotting positions for visually examining distributional model assumptions. For a single nucleation process and combining Equations (55) and (59), one obtains

(60) $$\ln\left[\frac{1}{1-\sum_{i=1}^{k}\frac{n_{i}\left(T_{i}\right)}{N_{0}}}\right]≅\frac{C_{1}}{\eta \left(T\right)J\left(T_{max}\right)}\exp\left(-\frac{C_{2}}{T{\left(\Delta T\right)}^{2}}\right)\;.$$

With Equations (47) and (50), we can replace $J(T_{max})$ by $J\left(T_{max}\right)=\left|q\right|/V{\left(\Delta T\right)}_{q}$. Finally, rearranging above relation and taking the logarithm, leads to

(61) $$\ln\left\{\frac{\eta (T)|q|}{{\left(\Delta T\right)}_{q}}\ln\left[\frac{1}{1-\sum_{i=1}^{k}\frac{n_{i}\left(T_{i}\right)}{N_{0}}}\right]\right\}\;≅\ln\left(C_{1}V\right)-\frac{C_{2}}{T{\left(\Delta T\right)}^{2}}\;.$$

For a plot of the left hand side of Equation (61), $\ln(\eta |q|H\left(T\right)/{\left(\Delta T\right)}_{q})$, against 1/$T{\left(\Delta T\right)}^{2}$, the data should line up as approximately a straight line going through the intercept $\ln\left(C_{1}V\right)$ with a slope $C_{2}$. In fitting the data, we replace ${\left(\Delta T\right)}_{q}$ for comparison also by the current undercooling, (ΔT).

## 3. Experimental Data and Their Interpretation

### 3.1. Brief Description of the Experiments

In the present study, we apply above-described methods to heterogeneous crystallization either at constant temperature or at cooling with a constant rate. In the experiments, the nucleation temperature, $T_{n}$, is reached by cooling starting from temperatures above the melting temperature, $T_{m}$ (in order to equilibrate the liquid and to remove, as far as possible, all eventually existing crystallites in it) in a particular experiment using an isothermal (upon rapid quenching to $T_{n}$) or isochronal (constant cooling rate, *q*) time-temperature protocol. In these experiments, either the average waiting time, ${\langle \tau \rangle }_{q}$, respectively, the average achievable undercooling, ${\langle \Delta T\rangle }_{q}$, required for the evolution of the first crystallite will be determined. The procedure requires a sufficiently frequent repetition of each of the time-temperature protocols due to the random nature of crystallization. As a particular example, we analyze liquid-to-crystal heterogeneous nucleation of supercooled lithium disilicate glass-forming melts. The details of the experimental procedure and the obtained results can be traced in [35,37].

### 3.2. Nucleation at Constant Temperature

**Figure 2:**
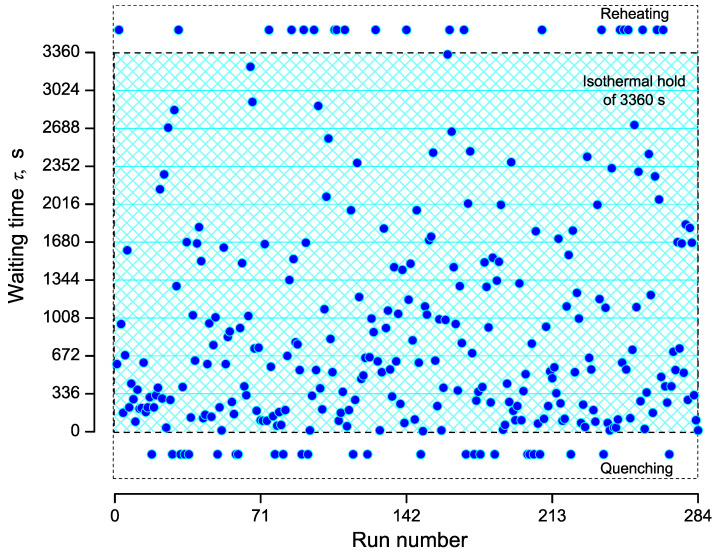
Waiting times, τ, in dependence on repetition (run number). The total number of runs equals $N_{0}=284$. Note that negative waiting times (τ<0) and waiting times (τ>3360 s) are included (and in turn virtually set as τ=−200 s and 3560 s) to highlight runs, which showed quenched-in crystals (crystallization occurred during quenching to $T_{n}$) and the absence of crystals within the holding time (crystallization occurred upon reheating), respectively.

A set of data for the time of formation of the first critical nucleus is shown in Figure 2. It corresponds to an isothermal time-temperature protocol with $T_{m}=1306$ K, $T_{n}=1173$ K, $\Delta T=T_{m}-T_{n}=133$ K. The total time of observation of nucleation at the given nucleation temperature was taken equal to $t_{n}=3360$ s, the number of runs equals $N_{0}=284$ (see Krüger et al. [37]). Figure 2 shows that 26 runs exhibit quenched-in crystals (τ<0) and 21 runs retain the sample to be amorphous within the holding time ($t_{n}>3360$ s). Within the isothermal hold, 237 runs led to crystallization.

**Figure 3:**
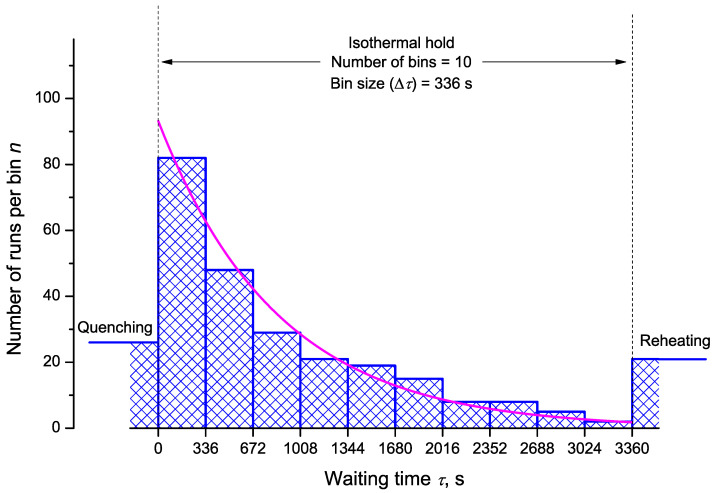
Histogram of number of runs crystallizing in the interval τ+Δτ with a bin size, Δτ=336 s. Note that outside the trail period (isothermal hold of $t_{n}=3360$ s) 26 runs crystallized during quenching and 21 during reheating. The full smooth curve in the figure, Ω=Ω(τ), is drawn utilizing Equation (13). Here λ is taken equal to $\lambda =0.00115\;s^{-1}$ as obtained via Equations (62) and (63).

Figure 3 shows the discrete probability distribution for a selection of 10 bins (bin size Δτ=336 s) within the isothermal hold of $t_{n}=3360$ s. The results are compared with the theoretical predictions, Ω=Ω(τ), as given by Equation (13). To calculate the mean time for the evolution of the first crystallite, only runs within the holding times are considered and with Equation (14), we obtain for the particular example under consideration

(62) $${\langle \tau \rangle }_{ss}=\frac{\left(82\times 168\;s)+(48\times 504\;s)+\ldots +(2\times 3192\;s\right)}{237}=851.3\;s\;.$$

Equations (18) and (32) yield

(63) $$JV=\lambda =\frac{1}{{\langle \tau \rangle }_{ss}}=0.00117\;s^{-1}\;.$$

**Figure 4:**
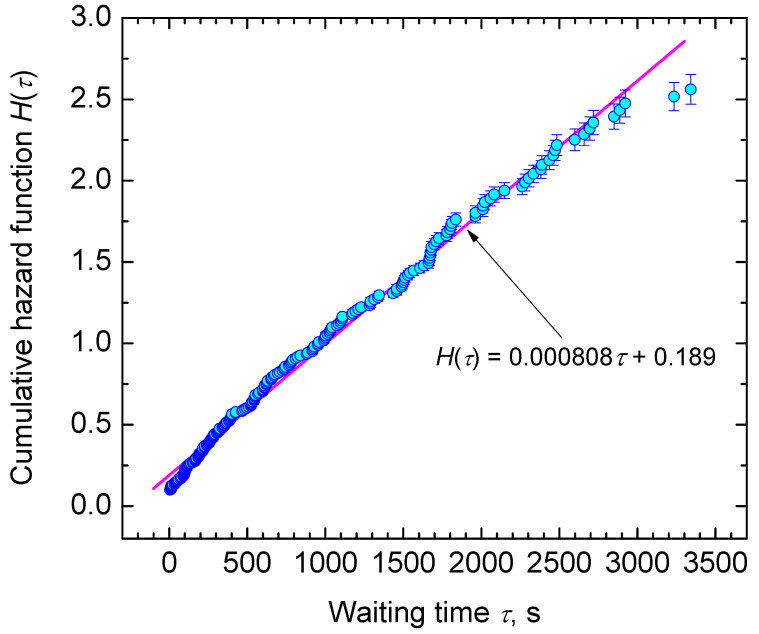
The cumulative hazard function, H(τ) (Equation (27)), in dependence on waiting time, τ.

In Figure 4, we show the cumulative hazard function, *H* (Equations (25)–(27), in dependence on time, τ. Note that, for *H* (Equation (27)), plotting positions of all runs are considered by sorting the intervals in ascending order from i=1,2,…,k (called their ranks) and summing up their values, $n_{i}/N_{0}$. Runs with τ<0 s and τ>3360 s are separated in this way as their ranks are ≤26 and >263. However, Figure 4 shows only those 237 runs, where critical cluster formation was observed within the trail period. All points line up almost as a straight line. Thus, it can be assumed that heterogeneous crystal nucleation is based on a single Poisson process, i.e., one type of sites at an external surface is active. The results do not rule out possible other nucleation processes at shorter or longer times, as only 83% of all runs are within the observation period. The best linear fit through the data is given by

(64) H(τ)=0.000808τ+0.189.

**Figure 5:**
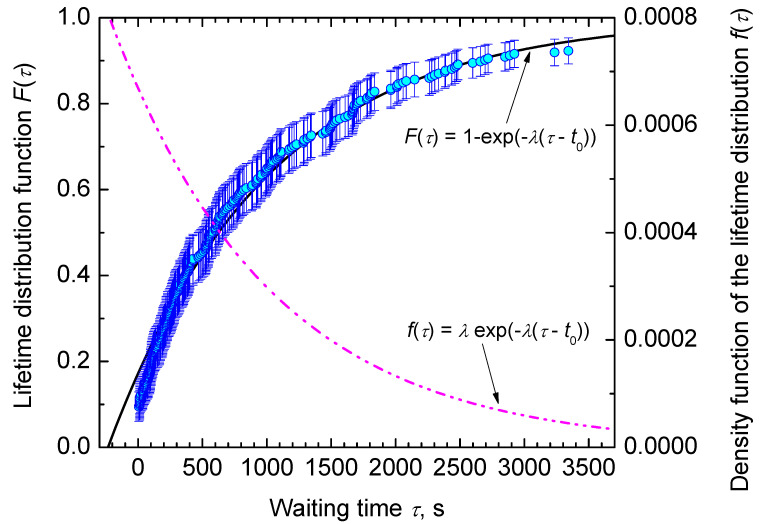
Lifetime distribution function, F(τ) or probability, $\tilde{P_{1}}\left(\tau \right)$ (Equations (19) and (21)) that at the moment of time, τ, the first critical cluster is formed in the system in the course of nucleation processes starting at τ=0. Simultaneously, also its derivative, the density function of lifetime distribution, f(τ) (Equation (23)), or the density of probability of formation of the first supercritical nucleus, ω(τ) (Equation (11)), is included. Both curves are drawn with a value of λ equal to λ=0.000808 and shifted on the time axis by $t_{0}=234$ s.

It leads to a value of λ=0.000808 in good agreement with Equation (63). As the first 26 ranks with values <0 are included into the analysis, the constant 0.189 is obtained. With this value of λ, we can determine the probability, $\tilde{P_{1}}\left(\tau \right)$ (or lifetime distribution function, F(τ) (cf. Equations (19) and (21)) that at the moment of time, τ, the first critical cluster is formed in the course of nucleation processes, starting at τ=0. In order to still allow an assessment of runs, which showed nucleation during quench (τ<0), we introduce the time shift, $t_{0}$, and obtain for it a value of −234 s from Equation (64). It is shown in Figure 5 that the theoretical and experimental data coincide widely.

### 3.3. Nucleation at Cooling with a Constant Cooling Rate

**Figure 6:**
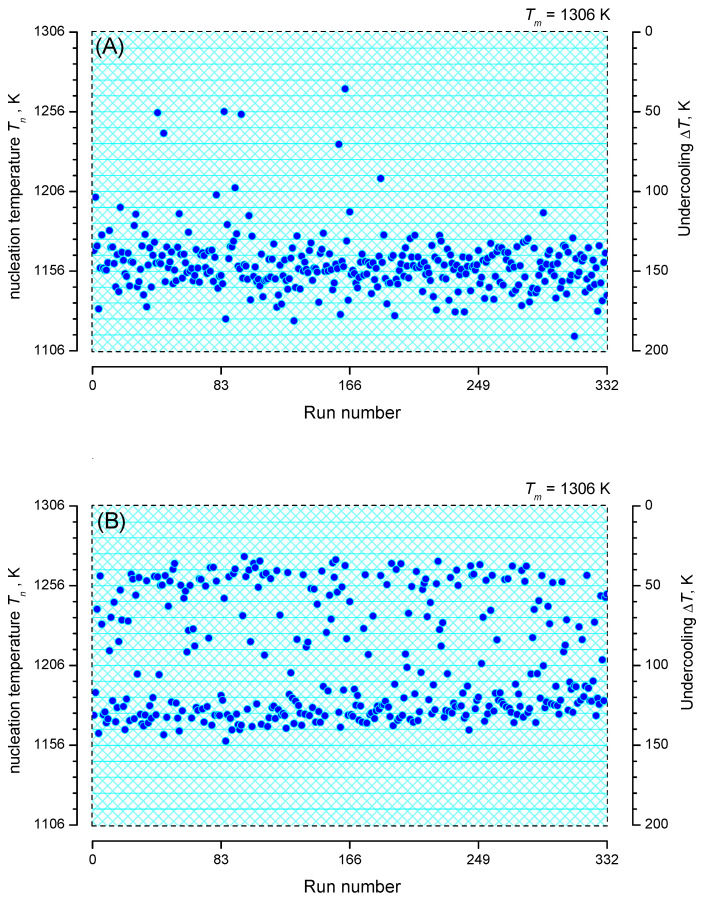
Nucleation temperatures, $T_{n}$, in dependence on repetition (run number) of the (**A**,**B**) sets of data of isochronal time-temperature protocols.

Analysis of the heterogeneous nucleation kinetics is exemplified by two sets of data as shown in Figure 6. Data set (A) of Krüger et al. [35] corresponds to an isochronal time-temperature protocol with $T_{m}=1306$ K, |q|=5 K min${}^{-1}$ and $N_{0}=332$, while data set (B) of Al-Mukadam [39] reflects $T_{m}=1306$ K, |q|=2.5 K min${}^{-1}$ and $N_{0}=332$. Figure 6 shows that in each run crystallization occurred during cooling down to 1106 K (no un-crystallized runs). Observation of nucleation was performed in the range 1270.4–1115.1 K and 1274.3–1158.5 K in the A and B data sets, respectively.

**Figure 7:**
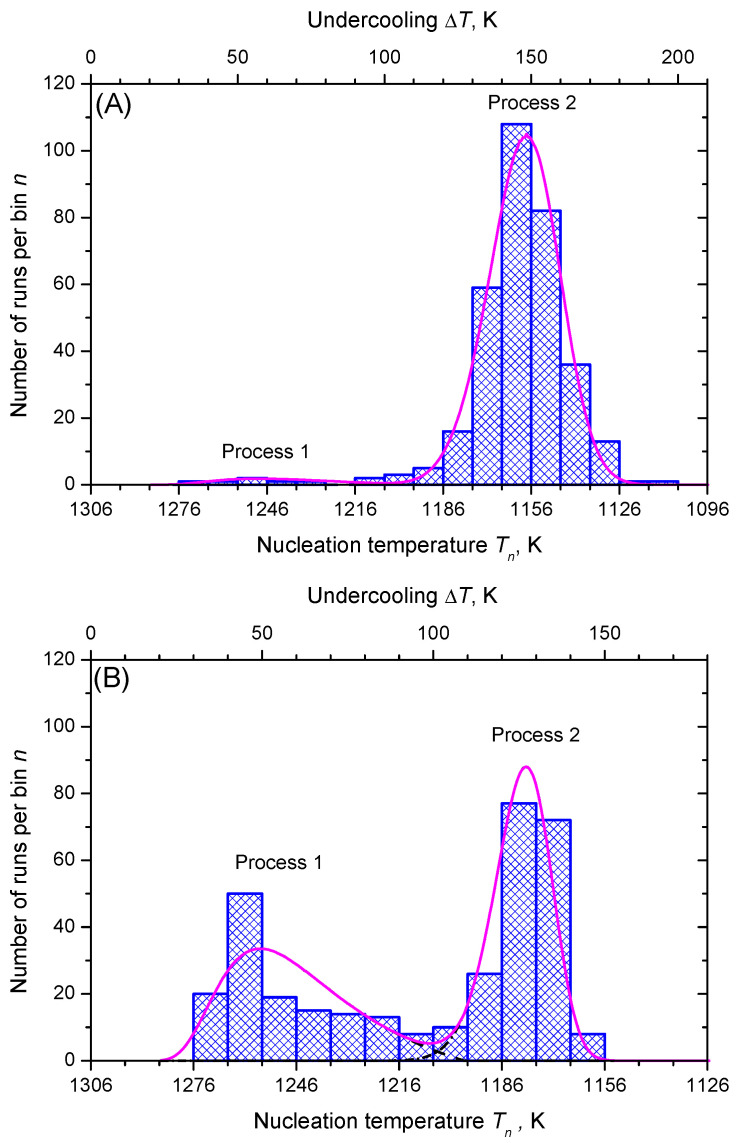
Histograms of the number of runs crystallizing in the interval $T_{n}+\Delta T$ with bin size, ΔT=10 K of the (**A**,**B**) sets of data. The full smooth curve in figure (**A**) is drawn utilizing Equation (61) and the values $\ln(C_{11}V)=-5.1$, $C_{21}=6\cdot 10^{6}$ K${}^{3}$ (process 1), $\ln(C_{12}V)=6.4$, $C_{22}=2.1\cdot 10^{8}$ K${}^{3}$ (process 2) and assuming that 10 runs can be assigned to process 1. For figure (**B**), the full smooth curve is drawn with $\ln(C_{11}V)=-2.8$, $C_{21}=5\cdot 10^{6}$ K${}^{3}$ (process 1) and $\ln(C_{12}V)=6.8$, $C_{22}=1.8\cdot 10^{8}$ K${}^{3}$ (process 2) and assigning 143 runs to process 1.

Figure 7 shows the discrete probability density distribution of nucleation temperatures, $T_{n}$, for selecting a bin size of ΔT=10 K with 15 and 12 bins, respectively. Two populations of nucleation events are evident: process 1 and, at lower temperatures, process 2. However, process 2 is more frequent in both sets of data.

**Figure 8:**
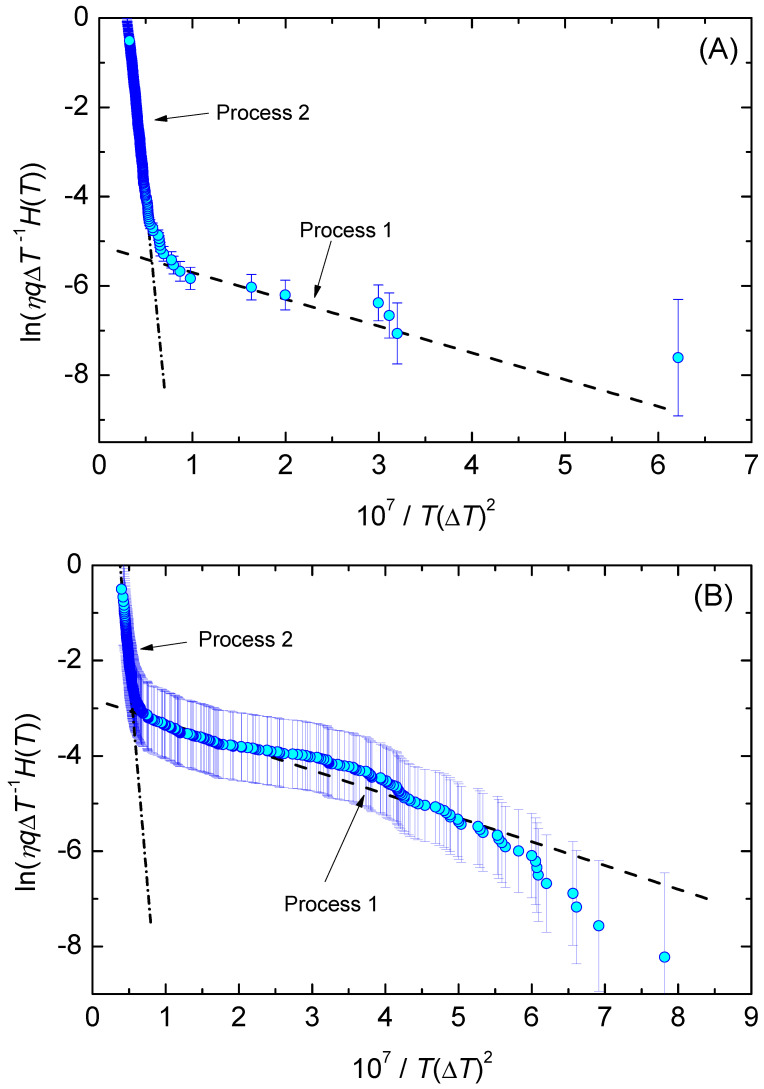
ln(η(T)|q|H(T)/ΔT) versus $1/T{\left(\Delta T\right)}^{2}$. For data set (**A**), the values $\ln(C_{11}V)=-5.1$, $C_{21}=6\cdot 10^{6}$ K${}^{3}$ (process 1, dashed line), $\ln(C_{12}V)=6.4$, $C_{22}=2.1\cdot 10^{8}$ K${}^{3}$ (process 2, dashed-dotted line) of Equation (61) are obtained by fitting straight lines through the respective sections of the data. For data set (**B**), the corresponding values are $\ln(C_{11}V)=-2.8$, $C_{21}=5\cdot 10^{6}$ K${}^{3}$ (process 1, dashed line) and $\ln(C_{12}V)=6.8$, $C_{22}=1.8\cdot 10^{8}$ K${}^{3}$ (process 2, dashed-dotted line).

**Figure 9:**
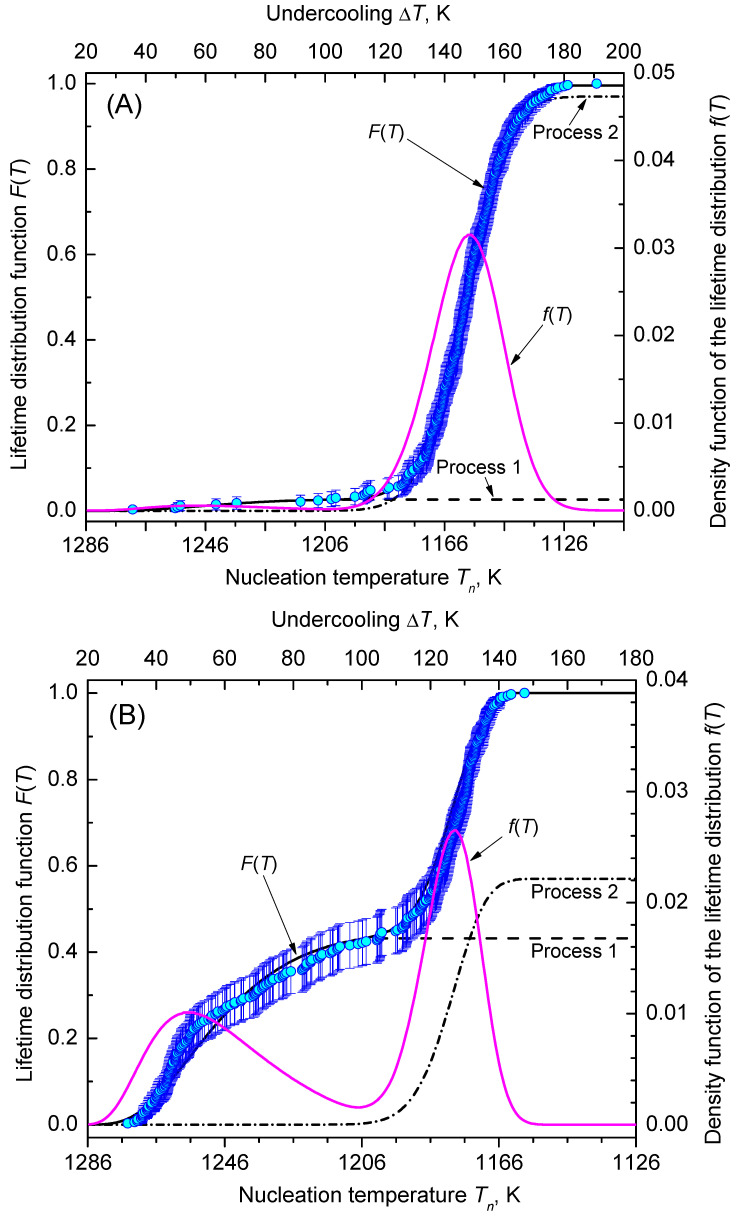
Lifetime distribution function, F(T), and the corresponding density function of the lifetime distribution, f(T). Dashed and dashed-dotted lines are the corresponding functions $F{\left(T\right)}_{1}$ and $F{\left(T\right)}_{2}$ (using the values given in Figure 8) of the nucleation processes 1 and 2, respectively, sharing the cumulative lifetime distribution. The best match with the plotting positions is achieved in (**A**), for the percentiles ∼3% (process 1, dashed line) and ∼97% (process 2, dashed-dotted line), whereas in (**B**), the percentiles are ∼43% and ∼57% for process 1 (dashed line) and process 2 (dashed-dotted line), correspondingly.

**Figure 10:**
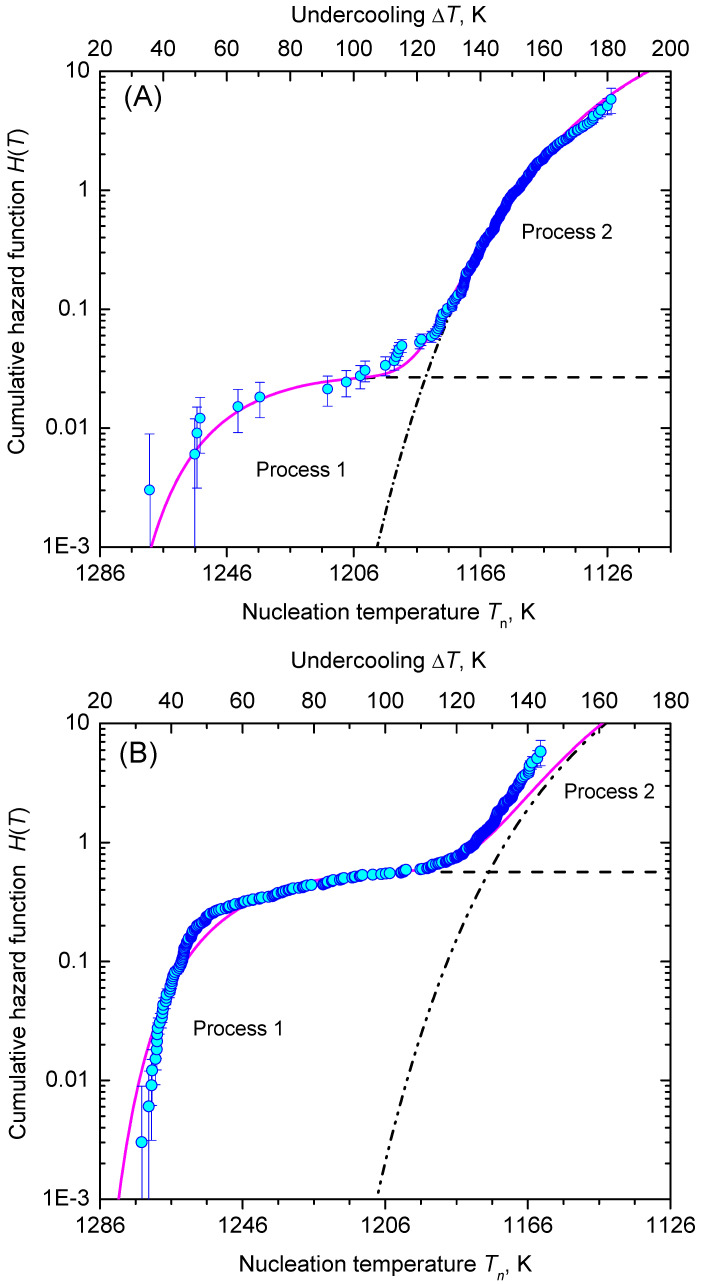
Cumulative hazard function, H(T), for the two data sets (**A** and **B**) considered. Dashed and dashed-dotted lines are the corresponding functions $H{\left(T\right)}_{1}$ and $H{\left(T\right)}_{2}$ (calculated using the values and percentiles given in Figure 8 and Figure 9, respectively) of the nucleation processes 1 and 2 sharing the cumulative hazard function. The full magenta line represents the sum of $H{\left(T\right)}_{1}$ and $H{\left(T\right)}_{2}$.

**Figure 11:**
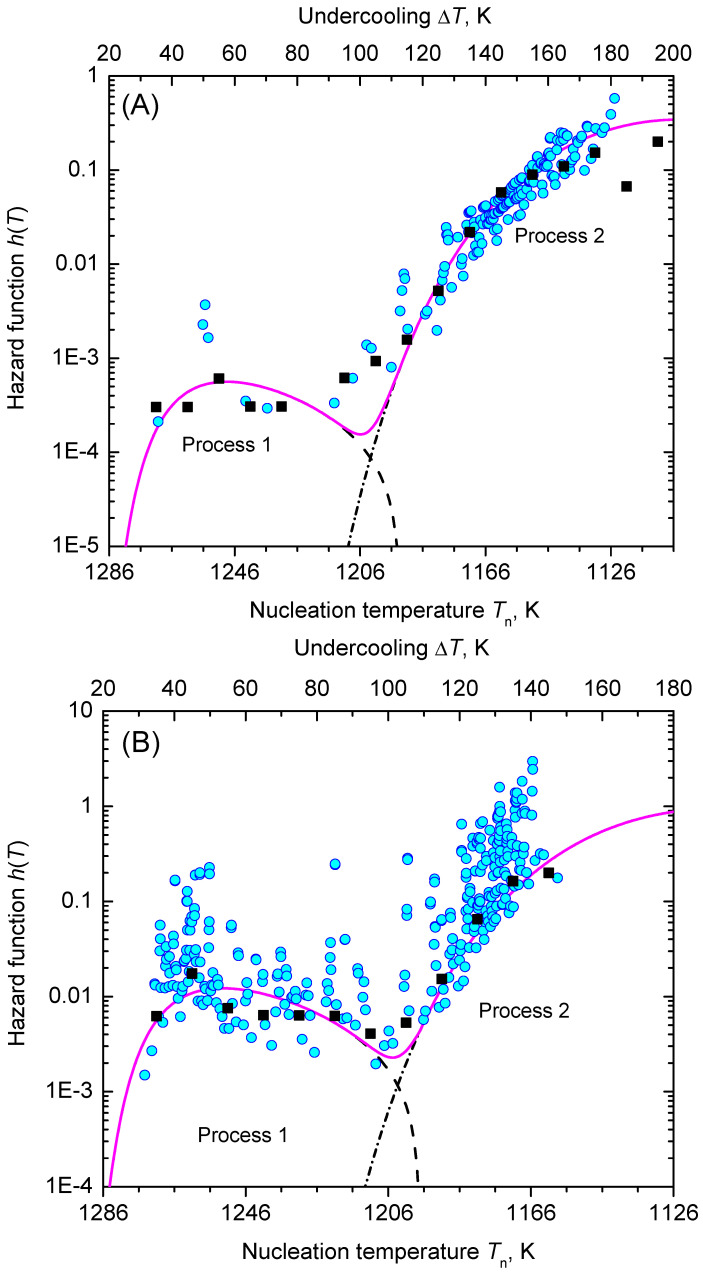
Hazard function, h(T), for the two data sets (**A** and **B**) considered. Dashed, dashed-dotted, and full lines correspond to the hazard functions of the nucleation processes 1, 2, and 1 + 2, respectively. Black squares are the estimated mean values of the hazard rate of each bin of Figure 7 computed according to the method of Gehan [75].

**Figure 12:**
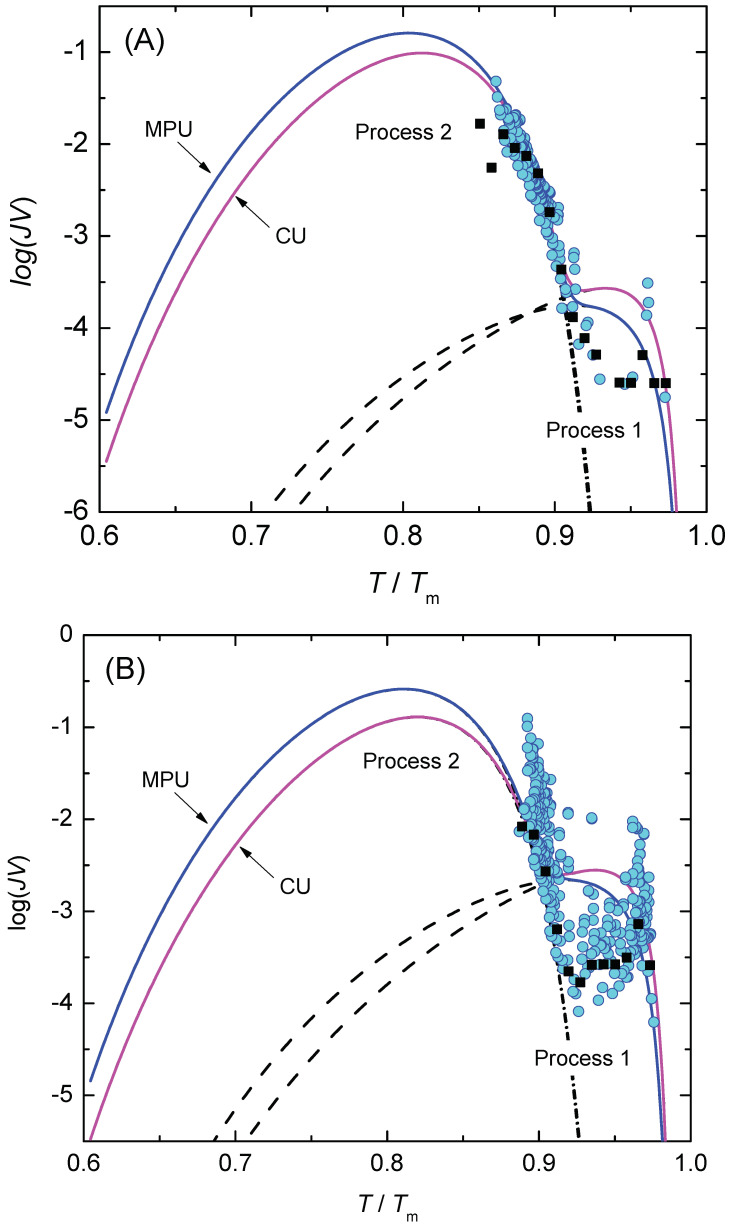
Tammann plot [17,62] of the crystal nucleation rates for the two data sets (**A**,**B**) considered. Dashed, dashed-dotted and full lines correspond to the nucleation processes 1, 2, and 1 + 2, respectively. CU indicates the fit of Equation (61) using the current undercooling ΔT, while MPU indicates fit of Equation (61) using the most probable undercooling, ${\left(\Delta T\right)}_{q}$. The estimated mean value of the hazard rate, h(τ), of each bin of Figure 7 (black square) computed according to the method of Gehan [75] is shown for comparison.

In Figure 8, we show the function, ln(η|q|H(T)/ΔT) (Equation (61)), is dependent on the inverse temperature scale, $1/T{\left(\Delta T\right)}^{2}$. The data points line up in two sections almost as straight lines, which are assigned to the nucleation processes 1 and 2, respectively. Figure 9 shows the lifetime distribution function, F(T), and the corresponding density function of the lifetime distribution, f(T). Figure 10 shows the cumulative hazard function, H(T), and the sharing of the two nucleation processes. In turn, the hazard function, h(T), is shown in Figure 11. We additionally present the hazard rate of each bin of Figure 7 as estimated by the method of Gehan [75]. This easy-to-apply method has been used in lifetime analysis of crystal nucleation data previously [22,23,24] to estimate the mean hazard rate of each interval (bin) from dividing the number of crystallized runs through the average number of non-crystallized runs (survivors) at the mid-point of the interval. Finally, in Figure 12 we show the rate curves of the two temperature-dependent crystal nucleation processes using the Tammann [17,62] diagram style, i.e., JV is plotted in logarithmic scales versus reduced temperature, $T/T_{m}$.

## 4. Results and Discussion

The application of probability concepts to crystal nucleation in glass-forming liquids is based on the description of the risk that in a certain volume of an undercooled liquid crystals nucleate and the accumulation of that hazard over time. Hereby, the hazard function (also named as hazard rate), *h*, measures the propensity to nucleate depending on the undercooling, ΔT, reached (isochronal condition) or the time, τ, waited (isothermal condition). It thus plays the key role in characterizing the process of crystal nucleation and in classifying lifetime distributions. To build the hazard function of a nucleation process, the lifetime distribution function is formed first in an easy-to-access step, for which we applied the natural estimator (Equation (19)) [76] forming the familiar staircase empirical lifetime function with an upward step of size $1/N_{0}$ at each τ or ΔT. The natural estimator is commonly known in survival statistics as the Kaplan–Meier estimator [77]. Pointwise confidence intervals (95%), for the estimated lifetime probabilities, are obtained using Greenwood’s variance formula for the inverse survival function [78] and approximating a two-sided (1−α) confidence interval. The experimental data are analyzed based on the assumption that heterogeneous crystal nucleation is based on a single homogeneous Poisson process (Figure 5) and on double non-homogeneous Poisson processes (Figure 9), respectively. Inspection of these figures shows that the fitted lifetime distribution functions are within the confidence intervals for almost all data points, but some exceedance is noticed for short waiting times in case of the isothermal data set.

**Figure 13:**
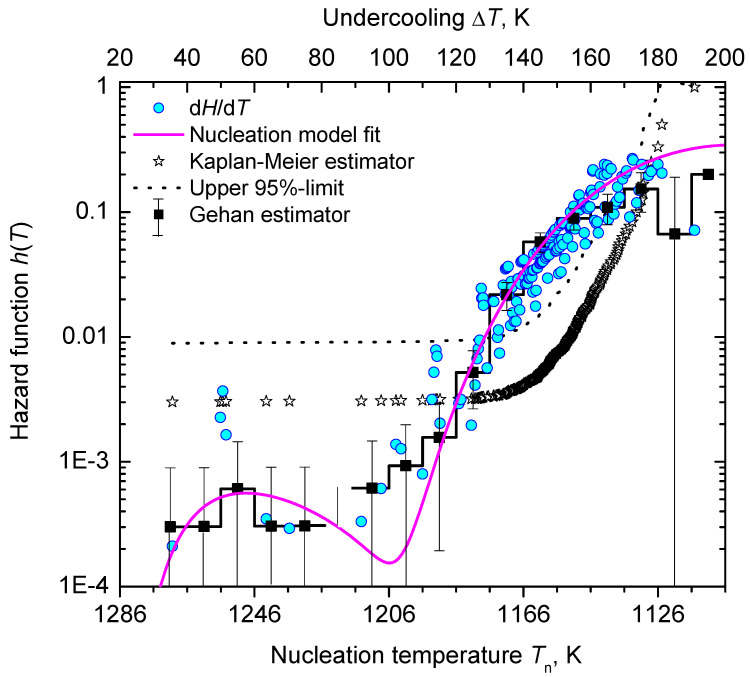
Comparison of estimates of the hazard function for nucleation at cooling with a constant cooling rate (dataset A). Kaplan–Meier estimator: for the sake of clarity, the pointwise upper 95% confidence limit is shown as a dotted line. The lower 95% confidence limit is <0 for all data points and not shown. Gehan estimator (bin size = 10 K): the pointwise 95% confidence interval is shown as error bars. Difference quotient (labeled as dH/dT): points are shown using the Kaplan–Meier estimator (Equation (27)) for $\tilde{H}$.

In the second step of the applied analysis, the plotting positions of the cumulative hazard function are estimated. Again, we used the natural estimator built upon the previously estimated lifetime function (see Equations (20) and (27)). Pointwise variances are computed using the formula provided by Rinne [79]. Figure 4 and Figure 10 show that the fitted crystal nucleation processes are well positioned within the corresponding confidence intervals. However, some compromises on the quality of the fits can be observed in Figure 10 for the dataset B. On the one hand, improvement can be gained by allowing a subdivision into more than two nucleation processes, as one may suggest from the inspection of the linearized cumulative hazard of Figure 8B. On the other hand, further subdivision reduces the number of data points per process and, with it, decreases the statistical significance of the nucleation parameters gained from this approach. In such a situation, the increase of the total number of runs on trail $N_{0}$ from experimental side is quite beneficial. Further, it should be noted that, for the linearization of the cumulative hazard function, a basic model for the temperature dependence of the nucleation rate was utilized (see Equation (59)). Modifications of this relation will be advanced in the near future and should account for more correct expressions for the thermodynamic driving force [73], the decoupling of diffusion and viscosity [74], and the assignment of different site activities of concurrent heterogeneous nucleation processes [11,12,46]. Furthermore, it should be stressed that direct estimates of the hazard rate from lifetime data were less likely to integrate successfully. Figure 13 shows exemplarily that the Kaplan–Meier estimate of *h* can be perceived only ineffectually in its function as the key parameter of describing the underlying nucleation rate in comparison with the difference quotient of the cumulative hazard and the nucleation model fit from the linearization of the cumulative hazard via Equation (61).

Alternatively, statistical analysis can start from the density distribution of nucleation events for estimating the lifetime and hazard functions. This approach, which was initially used in population census and death statistics and known as the life table method, can be of great value if all events are occurring within the trial period and the total number of events, $N_{0}$, is not too small. However, a life table of crystal nucleation requires a divided time or divided temperature axis. In consequence, estimates of the hazard function lack smoothness and continuity as the events are collected within each interval (bin) with the bin size as a possible confounding factor. Despite these drawbacks, Figure 13 shows that the results obtained using Gehan’s estimator [75] (for a bin size of 10 K) and the differentiated cumulative hazard data coincide widely. In turn, it must be noted that from inspection of Figure 13 neither Gehan’s nor Kaplan–Meier’s method of directly estimating the hazard function, *h*, seems to be an adequate option to discover concurrent nucleation mechanisms. In such cases, the described route via a linearized representation of the cumulative hazard, i.e., ln(Hηq/ΔT) vs. $1/T{\left(\Delta T\right)}^{2}$ (see Figure 8), can be very effective to provide such a conclusive evidence.

## 5. Conclusions

Due to the stochastic nature of the crystal nucleation process in glass-forming liquids, determination of kinetic parameters relies on the analysis of a large number of events. Thus, probabilistic concepts must be implemented to understand the variation in the achieved τ or ΔT from run to run if experiments at shallow undercooling are carried out. It is shown that the statistical methods of lifetime analysis can be of great value, as the natural estimate of the *H*-function can be computed in an easy-to-access step from the ranked lifetimes. In case of homogeneous Poisson processes (isothermal condition), a linear H(τ)-function indicates a single nucleation process and allows to directly derive the steady-state nucleation rate, JV, from its slope, whereas for a non-homogeneous Poisson process (isochronal condition) a linear representation of H(ΔT) is only achieved if a (classical) model for the temperature-dependent nucleation is applied. For the latter case (nucleation at cooling with a constant cooling rate), it is emphasized that the occurrence of more than one linear segment within the trial period indicates concurrent nucleation mechanisms with different $J(T/T_{m})V$-characteristics as highlighted by plots in the Tammann diagram style presented.

## Data Availability

The data presented in this study are available on request from the corresponding author.
